# Dentists’ perceptions of practicing patient-centred care: a qualitative exploration guided by the theoretical domains framework

**DOI:** 10.1186/s12903-026-08288-5

**Published:** 2026-04-06

**Authors:** Paige Cunnington, Heather Buchanan, Koula Asimakopoulou

**Affiliations:** 1 The Healthcare Improvement Studies Institute, Department of Public Health and Primary Care, University of Cambridge, Cambridge, CB1 8RN UK; 2 School of Medicine, Faculty of Medicine and Health Sciences, University of Nottingham, Nottingham, UK; 3 Faculty of Dentistry Oral and Craniofacial Sciences, Kings College London, London, UK; 4 Oxford Brookes University, Oxford, UK

**Keywords:** Dentistry, Patient-Centred Care, Qualitative, Theoretical Domains Framework

## Abstract

**Background:**

There has been a growing interest in adopting patient-centred care into dental practice. However, there lacks a consensus of what patient-centred care looks like within the dental context and dentists report several barriers to practicing it. This study explored dentists’ understanding of patient-centred care and is the first to use the Theoretical Domains Framework (TDF) to identify dentists’ perceived barriers and facilitators to practicing patient-centred care in private dental practice.

**Methods:**

Semi-structured interviews (*n* = 9) were conducted with dentists who completed ≥ 50% of their practice as a private dentist in the UK. Data were initially analysed using inductive thematic analysis to generate subthemes which were then deductively mapped to TDF domains.

**Results:**

Thirteen subthemes corresponded to eight TDF domains; ‘*knowledge*’, ‘*skills*’, ‘*beliefs about capabilities*’, ‘*optimism*’, ‘*beliefs about consequences*’, ‘*memory*,* attention and decision processes*’, ‘*environmental context and resources*’, and ‘*emotion*’. One additional subtheme (‘*Conceptualisation of patient-centred care*’) did not map onto any domain and reflected dentists’ perceptions about the components of patient-centred care.

**Conclusions:**

Findings revealed that whilst dentists’ understanding of patient-centred care aligns to academic definitions of patient-centred care, there remains a lack of consensus about patients’ responsibility for decision-making within a patient-centred approach. Furthermore, novel use of the TDF has identified several barriers and facilitators to patient-centred care which could be addressed to support the provision of patient-centred care in dental practice. Indeed, dentists may benefit from training in patient-centred care and future research should explore how establishing a patient-centred dental practice can facilitate patient-centred care.

**Supplementary Information:**

The online version contains supplementary material available at 10.1186/s12903-026-08288-5.

## Background

Patient-centred care (PCC)^1^ is care that adopts a biopsychosocial perspective to explore how patients experience illness [1] and the impact this has on their life [2]. Furthermore, PCC consists of developing a practitioner-patient relationship [2], involving the patient in their care and reaching a shared understanding of the aims and outcomes of treatment [1]. Within the United Kingdom (UK), it is an expectation that healthcare services provide PCC [3] as it can lead to increased patient wellbeing and satisfaction [4].

Whilst research has predominantly focused on PCC in medical settings, there has been a growing interest in how PCC is conceptualised and applied in dental practice [5–10]. Indeed, principles of PCC feature within the General Dental Council standards of care [11] and The Safe Practitioner framework for dental education [12]. Adopting PCC into dental practice could reduce patient litigation [13], build trust between dentists and patients [14] and facilitate dental practices to become focused on the prevention and management of diseases [15]. Moreover, it is necessary to explore PCC specifically within dentistry given that there are differences between medical and dental consultations that could affect the transferability of findings from medical to dental settings. For example, during dental consultations, patients have limited ability for verbal communication and treatment can be performed at the same time as diagnosis [16].

Initial research demonstrates that it is unclear the extent to which PCC has been implemented into dental practice [8]. In addition, there lacks a consensus of what PCC means for dentistry [5, 17–19] and the understanding of PCC by dentists differs to academic conceptualisations. Indeed, a model of how PCC could be applied to dentistry proposes that PCC involves exploring illness from a biopsychosocial perspective, developing a long-term dentist-patient relationship, reaching a shared understanding of the condition, and providing information and choice to patients to support them to make the final decision about their care [20]. In contrast, dentists thought that PCC makes patients feel involved in their care [19] and that a true partnership is one where the patient cooperates with the dentists’ advice [21]. Moreover, it is not widely accepted that patients can be in full control of their care [8] and dentists said that they allowed patients to be involved in decision-making, which is not representative of the equal partnership between dentist and patient encouraged by a patient-centred approach [17].

Research has also identified several barriers to practicing PCC within dental practice, including that it is time-consuming [6, 17, 21, 22] and there is a lack of formal training in PCC [19] leaving dentists feeling unprepared to address patients’ emotions [22]. Furthermore, some patient factors are barriers to PCC including patients with dental anxiety who do not want to engage in treatment discussions [21], patients who are too engaged in the consultation [17] and patients who are not willing to engage in the consultation [17, 21]. Patients’ lack of trust and stereotypes that dentists are money-oriented could also affect patients’ involvement [21]. Finally, patients’ education and socioeconomic background can affect dentist-patient communication [21] and patients’ understanding could limit their ability to make decisions [6].

Taken together, research demonstrates that there lacks a consensus about what PCC means for dentistry and there are several barriers to its’ delivery. However, this might not generalise to all dentists in the UK. First, studies have included dentists involved in teaching at undergraduate level [6, 13, 19] or NHS dentists [21], therefore findings may not generalise to dentists in private practice [21]. It would be beneficial to explore PCC amongst dentists practicing privately given the prevalence of private dentistry in the UK. Indeed, private and mixed dental practices comprise 15% and 30% of independent dental practices in the UK [23] and many dentists report that they are likely to reduce their NHS commitments in favour of private care in the near future [24]. Second, one study was conducted in Canada [22] however dentists’ experiences of PCC in Canada might differ to those in the UK [13].

Consequently, there is a need for research exploring dentists’ understanding of, and perceived barriers/facilitators to, PCC in private practice in the UK. Such research could inform the development of interventions to support the provision of PCC within dental practice. It has been recognised that interventions should be based on theory [25] as they are more likely to be effective [26]. One useful theory for intervention development is the Theoretical Domains Framework (TDF) [27]. The TDF consists of 14 domains which cover a comprehensive range of factors influencing healthcare professionals’ behaviour [27], including factors that influence the implementation of evidence-based recommendations [28]. The TDF domains map onto the Capability-Opportunity-Motivation-Behaviour (COM-B) model of behaviour change [27] and the framework has been used to identify barriers to the implementation of clinical practice behaviours in dental settings [29]. However, the TDF has not previously been used to explore PCC within dental practice. It would be beneficial to adopt the TDF in a qualitative design to explore dentists’ experiences of practicing PCC as this is useful when there is little understanding about the behaviour [28].

Therefore, the aim of this study is to explore dentists’ perceptions of practicing PCC in private dental practice in the UK by: (1) exploring the understanding of PCC amongst dentists practicing privately and (2) identifying what dentists perceive to be the barriers and facilitators to practicing PCC in private dental practice using the TDF.

## Methods

### Study design

This was an exploratory cross-sectional qualitative design, using semi-structured interviews with dentists working in private practice in the UK.

### Participants

To be eligible to participate, dentists had to complete ≥ 50% of their practice as a private dentist in the UK. Dentists from any specialty were eligible to take part in an interview. Given that recruitment of healthcare professionals can be challenging [30], several recruitment strategies were employed in this study. Similar to previous qualitative studies applying the TDF, a combination of purposive, snowball [31, 32] and convenience sampling were used. Dentists meeting the eligibility criteria were recruited via purposive sampling by: (1) contacting dental practices in the East Midlands, East of England and South East directly via email, in-person visits and messages over Facebook and (2) contacting the research team’s personal contacts who have contacts with dentists. Convenience sampling, by posting on a Facebook group for UK-based dentists and the researcher’s Twitter, and snowball sampling, by asking participants to share the study information with other dentists eligible to participate, were also employed to identify potential participants to take part in an interview. Dentists had the opportunity to be entered into a prize draw for a £50 voucher as an incentive to participate in the study.

### Procedure

This study was given ethical approval (reference: MEDS4008-23-03) and adhered to the British Psychological Society [33] Code of Human Research Ethics.

Recruitment began in April 2023. Dentists who were interested in participating from any recruitment strategy were either contacted by or asked to email the researcher and were provided with the participant information sheet. Dentists were only contacted if they had agreed to this. The researcher then emailed dentists who had agreed to participate to ask them to sign the consent form and to arrange a date/time for an interview.

One-to-one interviews were conducted over Microsoft Teams between June and August 2023. A holistic semi-structured interview schedule (Supplementary File 1) was developed, similar to Nowak et al. [21], to explore if dentists practice PCC, how they do this and their experiences of PCC (i.e., barriers and facilitators) in their private dental practice. At the start of each interview, dentists were asked to focus on their experiences of PCC in private practice and to answer some demographic questions about their age, gender, and career in private dentistry. Dentists were then asked to discuss their understanding of PCC and were shown two models of PCC, and asked to share their opinion on them: (1) a broad model [1] and (2) a model specific to dentistry [20]. Dentists were also asked questions based on all 14 TDF domains [27, 28], in addition to further concepts identified from the literature, to explore the barriers and facilitators to practicing PCC. The structure of TDF-based questions was adapted from previous research [34, 35]. Additional follow-up questions were asked throughout interviews based on dentists’ responses to explore their experiences in more detail. At the end of each interview, dentists were asked if they wanted to be entered into the prize draw and were read and emailed a copy of the debrief sheet.

Interviews were audio-recorded and transcribed verbatim by the researcher. Transcripts were checked for errors against the audio-recording and any identifiable information was anonymised. One pilot interview was conducted to ensure the questions were fit for purpose, but no changes were made to the interview schedule. Interviews lasted between 32 and 76 min (mean = 45 min). Interviews ended at the point of data saturation, which was when no new information was provided about the barriers and facilitators to practicing PCC [28].

### Analysis

Transcripts were first analysed inductively following Braun & Clarke’s [36] six steps of thematic analysis. First, transcripts were read repeatedly to allow for familiarisation with the data and notes of interesting features in the data were made. Second, the data were coded at the latent level to explore implicit meanings within the data related to dentists’ understanding of PCC and the factors affecting its delivery [37]. Third, codes were grouped into potential themes and sub-themes that represented the story within the data. Fourth, the whole dataset was read again to review how well themes represented the entire dataset and amendments were made until a final set of themes were generated. Fifth, themes were named, and a short summary of each theme was written. Finally, quotes were identified that best supported each theme. Themes were then mapped deductively onto TDF domains using a coding guideline [28] developed by the researcher that was based upon descriptions of TDF domains by Cane et al. [27] and tailored to the behaviour of practicing PCC. Conducting inductive analysis before deductive coding to the TDF allowed for an exploration of dentists’ understanding of PCC and for factors outside of the TDF to be identified, which is useful for exploratory research [38]. Indeed, PCC is under-explored in dentistry and there is uncertainty about what PCC means for dental practice, therefore this approach was appropriate.

## Results

Nine dentists (age range = 28–59 years) were recruited to participate in an interview: one through contacting dental practices over Facebook, two through personal contacts and six through snowball sampling. One participant had recently retired, however their responses were similar to other participants, therefore it was appropriate to include them in the study. An additional six dentists showed interest in the study however decided not to participate after receiving further information. Saturation was reached after nine interviews, as no new information about factors influencing the provision of PCC were identified. Participant characteristics are shown in Table 1.

**Table 1:** Participant characteristics

Characteristic	Total (*n* = 9)
Age (years)	
Mean (range)	52 (28–59)
Gender	
Male	8
Female	1
Years spent practicing as a private dentist	
Mean (range)	18 (3–25)
Dental Specialty	
General Dental Practitioner	7
Restorative Dentist	1
Dental Implants	1
Average time working in private practice per week (hours)	
Mean (range)	31 (22–40)

Fourteen subthemes about dentists’ understanding of and experiences practicing PCC in private dental practice were created through the inductive analysis. Thirteen subthemes were deductively mapped onto eight of the 14 domains of the TDF. One subtheme (‘Conceptualisation of PCC’) did not match to any TDF domain and is therefore presented separately. Codes in brackets represent participant number (P) and participant age (y).

### Conceptualisation of PCC

Dentists believed that PCC consists of several elements. To some, PCC involves working to patients’ “*best interests*” and is a holistic approach “*seeing the person behind the teeth*” (P1, 44y), by acknowledging interactions between general and oral health, and exploring socioeconomic factors that could affect patients’ care. All dentists emphasised the importance of developing a long-term dentist-patient relationship, by establishing rapport with patients and building trust:

“*the trust element has to be earned*” (P9, 59y).

Most dentists felt PCC involves reaching a common understanding, by exploring patients’ needs and expectations, and building their understanding of their health. Dentists highlighted that clinical discussions should be balanced, discussing the “*advantages*,* disadvantages*” (P5, 59y), and that information should be understandable. Dentists also emphasised that PCC involves sharing the responsibility for decision-making with patients, by giving them options and supporting them to make the final decision:

“*together we select an option that suits them the best*” (P3, 28y).

It appears that dentists’ beliefs about patients’ responsibility in PCC varies, with some recognising that patients can select treatments they would not choose whilst others find it challenging when patients want treatment they think is not in patients’ best interests:

“*If they’re gonna be making a harmful choice I would need to be leading them away from that choice*” (P2, 57y).

### Theoretical domains framework

TDF subthemes and illustrative quotes are shown in Table 2. A total of seven barriers, five facilitators, and one barrier and facilitator were identified as factors influencing dentists’ provision of PCC in private dental practice.

**Table 2:** TDF subthemes with quotes for practicing PCC in private dental practice

TDF Domain: Theme Title	Subtheme Title	Barrier /Facilitator	Quotes
Knowledge: Unfamiliar with the theory and introduction of PCC into practice	Unfamiliar with the theory and introduction of PCC into practice	Barrier	“Patient-centred care has only really been discussed in the last 4 or 5 years” (P4, 47y) “The medico-legal world has helped to make sure that [patient-centred care] that’s a very big focus as well” (P8, 57y)
Skills: Development of PCC skills	Lack of formal training in PCC	Barrier	“I can’t really remember any lectures at University about patient-centred care” (P1, 44y) “All these bits I guess I’ve had training or experience in or I’ve gone on courses on but it wouldn’t be termed person-centred care” (P6, 59y) “It would be very nice to have proper training in all this sort of stuff about what patient-centred care means” (P4, 47y)
	PCC skills are developed informally and by trial and error	Facilitator	“Most of it is through experience, learning through experience” (P9, 59y) “I feel that I’ve learnt a lot from the people that I work with” (P1, 44y) “It’s just purely reading” (P4, 47y)
	Verbal and non-verbal communication skills	Facilitator	“It’s always just been talking to the patient, listening to the patient” (P5, 59y) “We can explain it better to them through the use of photos” (P1, 44y)
Beliefs about capabilities: Confidence practicing and engaging in PCC	Confidence in own ability to practice PCC	Barrier & Facilitator	“I think I’m probably quite good at it” (P8, 57y) “I have it [PCC] and the other dentists have it” (P8, 57y) “It’s [PCC] that sort of background moral compass” (P2, 57y)
	Patients’ desire to get involved	Barrier	“Some patients will say oh I don’t want to know about that just do it, and that’s just not an approach you can take” (P2, 57y) “When you put the patient on a pedestal, and you can risk them leading the treatment plan which isn’t always appropriate because there are some things that patients don’t understand clinically that we do” (P3, 28y)
Optimism: PCC is underappreciated by some dentists	PCC is underappreciated by some dentists	Barrier	“That’s probably underappreciated at the moment I would say” (P6, 59y)
Beliefs about consequences: Perceived benefits of adopting PCC	PCC leads to a successful dental practice and better work environment	Facilitator	“I think it results in happier patients who are more satisfied with the outcome of treatment” (P8, 57y) “You also feel more comfortable performing treatment on someone that you’ve known regularly and for longer than a complete stranger who’s asking you to do X, Y, Z” (P3, 28y) “If you got on with people and you deliver person-centred care and something goes wrong, the patient is much more inclined to give you the opportunity to resolve the issue” (P6, 59y)
	PCC improves patients’ health	Facilitator	“I think it would have a really large positive clinical impact” (P3, 28y) “Patients that didn’t really need a whole amount of work done because they were looking after themselves which I think is the most important thing” (P7, 58y)
Memory, attention, and decision processes: Patients’ ability to engage in PCC	Patients’ ability to engage in PCC	Barrier	“The person making the decision, their options will be determined by how much they can spend” (P4, 47y) “Is dentistry available for them as individuals, does it fit in with their work schedule” (P6, 59y) “If there are shall we say cognitive issues such as dementia, autism, you know disabilities, then that can be very challenging” (P5, 59y)
Environmental context and resources: Establishing a patient-centred dental practice	Establishing a patient-centred dental practice	Facilitator	“I think by sitting down just a little bit away from the clinical environment it sets the tone for your relationship” (P6, 59y) “It’s not dictated by regulatory bodies it’s dictated by what the patient would like to have done” (P9, 59y)
Emotion: Managing emotions within the consultation	Patients’ anxiety becomes the focus of the consultation	Barrier	“I think sometimes that can come across patients just saying you just do what you want and just get on with it is actually a bit of anxiety” (P1, 44y) “There is a danger that the whole consultation or the whole appointment becomes centred around the needle phobia and not about other important issues to do with that patient” (P3, 28y)
	Managing own emotions when practicing PCC	Barrier	“If you’ve had family issues, arguments prior to work that might make you slightly less empathetic than normal” (P5, 59y) “There’s a whole bunch of external pressures that can influence how you approach things” (P2, 57y)

### Knowledge: unfamiliar with the theory and introduction of PCC into practice (Barrier)

Dentists appeared to lack knowledge about the theory of PCC as they lack consensus about when PCC was introduced into practice. Whilst some felt they have provided PCC throughout their career, others believed that PCC is a recent term that has been emphasised by medico-legal requirements. In addition, most dentists were unaware of models of PCC. Nevertheless, they felt that the models correspond to their practice, implying that current practice may include some components of PCC proposed by academic models.

“*Patient-centred care is just normal dentistry. I don’t think it’s anything particularly new*” (P5, 59y).

“*I haven’t seen them [models of PCC] formally before*,* but they strike a lot of cords with my own thinking*” (P8, 57y).

### Skills: development of PCC skills

#### Lack of formal training in PCC (barrier)

Dentists highlighted that there is a lack of training specific to PCC. Instead, PCC is an “*unsaid term*” (P2, 57y) in training. One dentist believed that PCC models correspond to what they’ve been taught to practice, suggesting that components of PCC are integrated within the dental curriculum though not collated under the term ‘PCC’. Nevertheless, dentists were open to receiving more training in PCC, indicating a need for formal instruction.

“*We never really received training that was specifically about patient-centred care*” (P3, 28y).

“*It would be very nice to have proper training in all this sort of stuff about what patient-centred care means*” (P4, 47y).

#### PCC skills are developed informally and by trial and error (facilitator)

Dentists appear to have learnt PCC skills informally throughout their career. By reviewing consultations and listening to patient feedback, dentists reflect on what has/has not worked to improve PCC skills. Experience also appears to be important for managing patient emotions, as many dentists recognised this is challenging as a younger practitioner. Some dentists also learnt PCC skills by reading and observing colleagues’ approaches.

“*I’ll learn a lot of that from the previous experiences and what has worked for you in the past*,* ok let’s keep doing that*” (P5, 59y).

“*I’m probably less comfortable*,* because I’ve been exposed to it a lot less*,* when this kind of emotional or personal issues that patients confide in me about*” (P3, 28y).

#### Verbal and non-verbal communication skills (facilitator)

Participants highlighted that good verbal and non-verbal communication skills facilitates PCC. Dentists indicated that listening and communicating with patients is key, and that non-verbal communication skills help them to understand patients. Some dentists also strengthened their communication with patients by using photographs as visual aids to help build patients’ understanding.

“*Not just listening to what they say*,* watching how they say it as well*” (P7, 58y).

### Beliefs about capabilities: confidence practicing and engaging in PCC

#### Confidence in own ability to practice PCC (barrier & facilitator)

Dentists’ confidence practicing PCC varied. Most were confident delivering PCC as they saw PCC as an attitude, implying that they think it is an innate ability. They didn’t think of the term ‘PCC’ when providing care, instead they reported doing the right thing by treating patients as human beings with “*respect*”, “*empathy*” and “*being honest*” in a way they want themselves/others to be treated. In contrast, some dentists were less confident practicing PCC as they were unsure whether their understanding of PCC reflects what it is, therefore they were uncertain if they were practicing all elements of it.

“*I think it’s going back to that unknown unknowns*,* I know what I know and I do what I can*,* but I’m not 100% sure I’m doing everything I can because I don’t know what I should be doing*” (P4, 47y).

#### Patients’ desire to get involved (barrier)

Patients’ level of involvement in consultations limits PCC. Patients who don’t want responsibility and prefer the dentist to take charge of decision-making make PCC more difficult as the dentist is unwilling to do this. Conversely, one dentist found it challenging when patients have a high level of involvement as they lack the clinical knowledge to make decisions alone.

“*It’s very difficult to be patient-centred because the patients really been unwilling or unmotivated to take responsibility for their health*” (P3, 28y).

It also appears that patients’ involvement in decision-making varies depending on clinical need. Indeed, one participant indicated that patients’ desire to have less involvement in the consultation may not be a barrier in emergency consultations:

“*If I have a heart attack*,* I don’t really want the cardiologist to go through all the options*” (P6, 59y),

### Optimism: PCC is underappreciated by some dentists (barrier)

Most participants were advocates for PCC and had no concerns with the approach, believing that “*it’s a key component*” (P6, 59y). However, participants recognised that PCC is underappreciated by some dentists who do not see the value of PCC and do not view it to be as important as clinical skills. This suggests that implementing PCC into practice could be challenging as it may not be widely accepted.

“*A lot of people don’t see the benefit of patient-centred care*,* a lot of students wouldn’t go to those lectures so it’s kind of considered nowhere near as important as a good filling you know when it’s most totally the other way round*” (P4, 47y).

### Beliefs about consequences: perceived benefits of adopting PCC

#### PCC leads to a successful dental practice and better work environment (facilitator)

Dentists believed that PCC leads to a successful dental practice as patients are satisfied with their care, they feel valued, and they trust the dentist. Consequently, patients are loyal to the practice, returning for appointments and referring their dentist to others. Having a trusting cohort of patients also leads to a better work environment, as patients value dentists’ suggestions and are more inclined to allow them to rectify problems, indicating that PCC could reduce litigation. Furthermore, by building a dentist-patient relationship, dentists are familiar with patients therefore they feel comfortable providing treatment.

“*If you have a good rapport it will allow long-term success*” (P5, 59y).

“*If you got on with people and you deliver person-centred care and something goes wrong*,* the patient is much more inclined to give you the opportunity to resolve the issue*” (P6, 59y).

#### PCC improves patients’ health (facilitator)

Some dentists were confident that PCC leads to improvements in patients’ health as patients’ take responsibility for their health, regularly attend appointments and it makes “*behaviour change a bit easier*” (P4, 47y). This implies that PCC could motivate patients to improve their health behaviours.

“*I think any of the outcomes are gonna be improved with a person-centred approach*” (P6, 59y).

### Memory, attention and decision processes: patients’ ability to engage in PCC (barrier)

Dentists indicated that factors limiting patients’ ability to engage in consultations are a barrier to PCC. Some factors are outside of dentists’ control, yet they prevent dentists from providing PCC to some patients. Patients’ finances and availability may prevent them from having the best treatment for them or “*physical impediments to accessing the care*” (P7, 58y) can limit their access to the practice. Some dentists also highlighted that PCC is challenging with patients who cannot fully engage in decision-making (e.g., patients with dementia, limited English, young children) as informed consent discussions are restricted.

“*It’s kind of impossible to have that really informed consent discussion of pros and cons*,* different options with that kind of patient because there’s obviously an inherent language barrier*” (P3, 28y).

### Environmental context and resources: establishing a patient-centred dental practice (facilitator)

Dentists believed that a “*patient-centred work environment*” (P6, 59y), including the organisation and set-up of the practice facilitates PCC. They highlighted the importance of a patient-centred dental team, where everyone supports PCC, from discussing cases with colleagues to reflecting on consultations with dental nurses as they “*know the patients too*” (P1, 44y). There also appears to be more opportunity for PCC in private practice compared to the NHS. Whilst dentists recognised that PCC is time-consuming, they emphasised that there is more time for PCC in private practice. Moreover, dentists implied that they have more autonomy to provide care that is in patients’ best interests in private practice due to fewer regulatory and financial pressures.

“*I would say everybody in our practice*,* from receptionists*,* dental nurses*,* admin staff*,* all you know technicians*,* all need to contribute to patient-centred care*” (P1, 44y).

“*It’s much easier in private because you can spend the time and you can set the fee for all of that*” (P7, 58y).

### Emotion: managing emotions within the consultation

#### Patients’ anxiety becomes the focus of the consultation (barrier)

Dentists emphasised that PCC is more challenging with anxious patients as they might find it difficult to participate in the consultation. Dentists also recognised that patients’ anxieties can limit the progress of the consultation as their fear becomes the centre of discussions. Consequently, discussions about the patient’s oral health are restricted.

“*Patients’ emotions can impact on the quality of information gathering and the quality of communication that goes on*” (P8, 57y).

#### Managing own emotions when practicing PCC (barrier)

Some dentists believed that practicing PCC can occasionally be emotionally demanding. They emphasised that they are people too, therefore the emotional energy required to listen to patients’ emotions and past experiences can accumulate over time to become stressful. Some dentists also recognised that pressures in their home/work life can sometimes reduce their empathy during consultations. To prevent pressures from influencing consultations, dentists highlighted the need to “*compartmentalise*”.

“*most dentists probably hide their emotions*” (P4, 47y).

“*If you’re not patient-centred it’s probably less work and a bit easier emotionally from the dentists’ point of view*” (P1, 44y).

## Discussion

This study explored dentists’ experiences of PCC in private dental practice in the UK by exploring their understanding of PCC and identifying their perceived barriers and facilitators to practicing PCC using the TDF. Indeed, inductive and deductive analysis identified eight of the 14 TDF domains to be barriers and facilitators to practicing PCC: ‘knowledge’, ‘skills’, ‘beliefs about capabilities’, ‘optimism’, ‘beliefs about consequences’, ‘memory, attention and decision processes’, ‘environmental context and resources’, and ‘emotion’. An additional subtheme (‘Conceptualisation of PCC’) was generated but this did not correspond to any TDF domain.

Findings suggest that dentists in private practice may have some understanding of what PCC involves. Indeed, dentists believed that PCC involves seeing the whole person, building a dentist-patient relationship, reaching a common understanding, and sharing the responsibility for decision-making which reflects the elements of PCC proposed by Scambler and Asimakopoulou [20]. However, it appears that sharing the responsibility for decision-making is challenging when patients want treatment that dentists do not feel is in their best interests. This is consistent with previous research which demonstrates that it is not widely accepted for patients to be in control of their care [8] and patients have a choice so long as it is in their clinical best interests [19]. It has been suggested that the right decision in PCC is the one that the patient thinks is best for them, regardless of whether this is clinically correct [6]. Consequently, it appears that there remains a need to reach a consensus about the ideal level of responsibility that patients have for decision-making within dental consultations.

Consistent with previous research [19, 22], dentists reported that there is a lack of training in PCC. Consequently, dentists developed PCC skills in the workplace, consistent with findings by Scambler et al. [19]. Indeed, dentists learnt PCC skills through experience, and this was particularly important for being able to manage patients’ emotions during consultations. Dentists have previously reported finding it challenging to manage patients’ emotions due to a lack of training [22]. However, findings of this study suggest that training alone is insufficient to increase dentists’ confidence with patients’ emotions as this skill takes time and practice to develop. Findings from this study also suggest that reflecting on patient feedback helps dentists to develop PCC skills. Whilst this has been identified as useful amongst doctors [39], this appears to be a novel finding amongst dentists. Therefore, encouraging dentists to learn from patient feedback could help to increase dentists’ confidence in some elements of PCC. Nevertheless, whilst learning from experience supports dentists to practice PCC, formal training in PCC is still needed. This could improve dentists’ theoretical understanding and confidence practicing PCC. Training should be based on evidence-based principles, emphasise the importance of PCC for positive outcomes, include active learning such as role play or simulation [40] and be able to fit into a busy practitioner’s schedule (e.g. delivered in the workplace or online) [39]. This training could be delivered by Health Psychologists. However, findings suggest that ensuring attendance at training may prove challenging as some dentists underappreciate the value of PCC. Less positive attitudes towards PCC amongst dentists [16] and dental students [7] have previously been reported therefore it may be necessary for future work to improve dentists’ perception of PCC before delivering training.

The findings of this study also demonstrate that dentists believe there are some patient factors that affect PCC. Consistent with existing research [17, 21], findings revealed that anxious patients find participating in consultations difficult and patients’ under-involvement in consultations is a barrier to PCC. Whilst it is acceptable for patients to not engage in decision-making, this decision should be informed [41]. However, some participants felt uncomfortable making decisions for patients. This suggests that dentists may need support to engage in informed discussions to identify patients’ desired involvement. For example, dentists could use decision-aids as they help patients understand their role in decision-making and only add a few minutes onto the usual consultation length [42]. Findings also revealed that patients’ desire to participate in decision-making may vary depending on the purpose of the consultation and patients’ under-involvement may not be a barrier in emergencies. Indeed, previous research suggests that patients’ desired involvement depends on clinical need [43] and the presence of pain [44]. This decision should also be informed however there is limited understanding of decision-making processes in such situations therefore future research should explore this.

Previous research has also identified that patients’ education, socioeconomic background [21] and understanding [6] limit PCC. This was not found in this study, however participants felt that PCC involves exploring the socioeconomic factors that could affect care, therefore they may have been more comfortable around patients with varied understanding and socioeconomic backgrounds. Instead, dentists in this study found PCC challenging with patients who have limited ability to engage in informed consent discussions. Whilst previous research demonstrates that dentists find informed consent discussions challenging with patients with cognitive impairments [45] or language barriers [46], shared decision-making and informed consent are distinct concepts [47]. Consequently, patients’ limited ability to engage may be a barrier to informed consent rather than to PCC.

External pressures in dentists’ home/work-life are also a barrier to PCC as it reduces their empathy during consultations. This is unsurprising given that dentists’ empathy is important for PCC [18] and predicts patient-centred attitude [16]. Whilst a stressful work environment and stress have been found to decrease medical professionals’ PCC [48], this appears to be a novel finding amongst dentists. Consequently, there may be a need to provide support to dentists to manage home/work-life pressures to facilitate their provision of PCC.

Finally, findings revealed that the dental practice itself supports PCC. First, having a patient-centred dental team is important. This is understandable as previous research suggests that there should be good communication between the dental team and patients [49, 50] and dental hygienists should build patients’ trust [51]. However, it is unclear how PCC is understood by everyone in the dental team or what they perceive their role in PCC to be, therefore this should be explored in future research. Second, the layout of the dental practice can be set-up to support PCC. This may act as a form of non-verbal communication. Indeed, examination room characteristics are an important element of non-verbal communication in GP surgeries [52], therefore this may also be important for dental practices. It is unclear how non-verbal communication is used by dentists therefore future research should explore this. Finally, PCC appears to be easier in private practice compared to the NHS as there is more time, along with fewer regulatory and financial pressures. PCC is time-consuming [6, 17, 22], therefore NHS dentists think that limited time during consultations restricts PCC [21]. Moreover, dental targets within the NHS drive dentists to meet objectives rather than see patients as individuals so opportunity for PCC is limited [53]. Therefore, this study demonstrates that changes to the management of NHS practices may be necessary to facilitate PCC.

### Strengths and limitations

This study is the first to apply the TDF to explore PCC in dentistry and one of few [54] to do this across different healthcare contexts. Adopting the TDF in this study has identified several factors that influence dentists’ provision of PCC which could be addressed to improve the implementation of PCC into dental practice. In addition, when using the TDF the behaviour being studied should be specific about who needs to do it and what they need to do [28] however this could prove challenging when studying PCC given the lack of an agreed upon definition. Consequently, this study demonstrates that the TDF can be applied to behaviours which are difficult to specify. An additional strength of this study is that it applied the TDF in full, which is not commonly done within dental research. By using the TDF in its’ entirety it ensures that findings can be compared across studies [29]. Finally, this study conducted inductive analysis prior to deductive coding to the TDF which allowed for factors outside of the TDF to be identified [38].

There are several limitations to this study. First, one researcher coded data however this should be done by two researchers to increase reliability [28]. Nevertheless, as data were initially coded at the latent level, inter-rater reliability may not have been appropriate [55]. Second, most dentists interviewed had over 15 years’ experience therefore findings may not reflect the views of dentists with less experience. Indeed, provision of PCC might differ depending on experience [6], therefore future research should explore PCC amongst less experienced dentists. Third, many of the dentists interviewed were General Dental Practitioners however experiences of PCC may differ across different dental specialties. Finally, there could be a selection bias as dentists volunteered to participate [56], therefore their views about PCC may differ to dentists that did not participate.

## Conclusions

Overall, findings revealed that private dentists understand some components of PCC however there remains a lack of consensus about patients’ responsibility for decision-making in dental consultations. Furthermore, whilst findings revealed that PCC may be easier in private practice, dentists still face barriers to its delivery. Addressing these barriers could be useful for both NHS and private dentists. Indeed, training in PCC and emotional support should be offered to dentists. Future research is also needed to explore how a patient-centred team and non-verbal communication skills could support the provision of PCC in dental practice.

## Supplementary Information

Supplementary Material 1.

## Data Availability

The data are not publicly available as sharing this data could potentially compromise participant privacy. For further inquiries, please contact the corresponding author.

## Footnotes

1 Patient-centred care (PCC) is care that involves exploring how patients experience illness [1], building a practitioner-patient relationship [2] and reaching a shared understanding of the aims and outcomes of treatment [1].

## Abbreviations

PCC: Patient-centred care
TDF: Theoretical domains framework
