# Supplementary material for: Dentists’ perceptions of practicing patient-centred care: a qualitative exploration guided by the theoretical domains framework
Source: BMC Oral Health. 2026 Apr 6;26:953. doi: 10.1186/s12903-026-08288-5 (PMC13235094; doi:10.1186/s12903-026-08288-5)
Supplement: Supplementary file 1 — Supplementary Material 1. [file 12903_2026_8288_MOESM1_ESM.docx]

**Supplementary File 1: Interview Schedule**

This document contains the semi-structured interview schedule utilised in this study. Where questions were based on domains of the Theoretical Domains Framework (TDF), this is included in brackets next to the question.

**Interview schedule**

Q1: Please can you start by telling me about the concept of patient-centred care. What does this mean to you? (*TDF domain: knowledge*)

Q1.1: Please can you provide an example of what patient-centred care looks like in your consultations in your private practice?

Q2: Are you aware of these models of patient-centred care?

Model 1: patient-centred care [1]:

Patient-centred care is made up of 5 components:

- Biopsychosocial perspective: exploring the patients’ condition from a biopsychosocial perspective.
- The ‘patient-as-person’: exploring and understanding how the patient experiences the condition.
- Sharing power and responsibility: involving the patient in their care.
- The therapeutic alliance: reaching a shared understanding of the aims and outcomes from the treatment.
- The ‘dentist-as-person’: how the personal characteristics of the practitioner affect care.

Model 2: patient-centred care in dentistry [2]:

Patient-centred care is:

- Taking a holistic approach to understand the patients’ biopsychosocial circumstance and how illness affects their life.
- Developing a long-term relationship with the patient facilitating a common understanding of the problem, the goal of treatment and the role that the dentist and patient will assume.
- Patient is aware that they can choose the treatment that is suitable for them and they are supported in making this decision.

There are 4 levels for how patient-centred care can look like in dental practice:

- Level 1: providing information in a didactic format to the patient about their health.
- Level 2: level 1 with the patient made aware that they can choose between different treatment options.
- Level 3: supporting the patient to make an informed decision.
- Level 4: patients have the information, choice and support to come to an informed decision so they are responsible for making the ultimate decision about their treatment suited to their context.

Q3: Do these models represent what patient-centred care means to you?

Q3.1: Please can you tell me how they represent what patient-centred care means to you?

Q3.2: How do these models represent what patient-centred care means to you?

Q3.3: Do you think that one model corresponds to your understanding of patient-centred care more than the other?

Q3.4: Please can you tell me about why they don’t represent what patient-centred care means to you?

Q4: Please can you tell me about how patient-centred care sits within your responsibilities as a private dentist? (*TDF domain: social/professional role and identity*)

Q5: Please can you tell me about any training you have received in patient-centred care as a private dentist (*TDF domain: skills*)

Q5.1 How else have you acquired the skills to practice patient-centred care as a private dentist?

Q6: How confident are you in your ability to deliver patient-centred consultations? (*TDF domain: beliefs about capabilities*)

Q6.1: How confident are you to be able to address patients’ emotions? [3]

Q7: How do you think that a patient-centred approach could benefit your consultations? (*TDF domain: beliefs about consequences*)

Q7.1: How does a patient-centred approach affect the dentist-patient relationship?

Q7.2: How does a patient-centred approach affect the trust between yourself and the patient? [4]

Q8: Please can you tell me about any disadvantages that you think there are to a patient-centred approach for your consultations? (*TDF domain: beliefs about consequences*)

Q9: How confident are you that patient-centred care can improve patient outcomes? (*TDF domain: optimism*)

Q9.1: How confident are you that patient-centred care can help with the prevention of oral diseases? [5]

Q9.2: Please can you tell me about how this affects your approach to care?

Q10: Do you feel like you are making a difference when acting in a way that is patient-centred? (*TDF domain: reinforcement*)

Q10.1: Please can you give an example of this?

Q10.2: How does this affect how you practice patient-centred care?

Q11: Please can you tell me about the resources and support that you have to practice patient-centred care in your private dental practice? (*TDF domain: environmental context and resources*)

Prompt: time [3, 6-7]

Q11.1: How does this influence how patient-centred you are during your consultations?

Q11.2: How do you think you could have more support to be patient-centred in your private dental practice?

Q12: How often do you intend to be patient-centred in your consultations? (*TDF domain: intentions*)

Q12.1: Please can you tell me about circumstances where you do intend to take a patient-centred approach?

Q12.2: What about those where you do not intend to take a patient-centred approach?

Q13: How do you focus your attention on being patient-centred during your consultations? (*TDF domain: memory, attention and decision processes*)

Q14: How often does something else take priority over being patient-centred? (*TDF domain: goals*)

Q14.1: Please can you tell me about why this is more important than being patient-centred?

Q15: How do your emotions influence how patient-centred you are during your consultations? (*TDF domain: emotion*)

Prompt: stress, burn-out [8]

Q16: How do your patients’ emotions influence how patient-centred the consultation is?

Q16.1: Please can you tell me about patient-centred care with anxious patients? [7]

Q16.2: Please can you provide an example of this and what you do in this situation?

Q17: How does a patients’ willingness to be involved in the consultation affect your approach to patient-centred care? [6]

Q18: Please can you tell me about other patient factors that you think affect patient-centred care?

Prompt: socioeconomic status and education [7]

Q19: Please can you tell me about how your colleagues and other dentists in private dentistry practice patient-centred care? (*TDF domain: social influences*)

Q19.1: How does this influence how patient-centred you are in your consultations?

Q20: Please can you tell me about how you reflect on the implementation of a patient-centred approach into a consultation? (*TDF domain: behavioural regulation*)

Q20.1: How do you reflect on how successful this was?

Q20.2: How does this reflection affect patient-centred care in future consultations?

21. Overall, how do you feel about patient-centred care in private dental practice?

**References**

[1] N. Mead, P. Bower, Patient-centredness: a conceptual framework and review of the empirical literature, Soc Sci Med. 51 (2000) 1087–1110. <https://doi.org/10.1016/S0277-9536(00)00098-8>.

[2] S. Scambler, K. Asimakopoulou, A model of patient-centred care – turning good care into patient-centred care, Br Dent J. 217 (2014) 225–228. <https://doi.org/10.1038/sj.bdj.2014.755>.

[3] N. Apelian, J.-N. Vergnes, C. Bedos, Is the dental profession ready for person-centred care?, Br Dent J. 229 (2020) 133–137. <https://doi.org/10.1038/s41415-020-1650-3>.

[4] S. Yuan, D. John, S. Shambhunath, G. Humphris, A scoping review to explore patient trust in dentistry: the definition, assessment and dental professionals’ perception, Br Dent J. (2023). <https://doi.org/10.1038/s41415-023-5882-x>.

[5] S. Alrawiai, K. Asimakopoulou, S. Scambler, Patient‐Centred Care in Dentistry: Definitions and Models ‐ Commentary, European Journal of Dental Education. 25 (2021) 637–640. <https://doi.org/10.1111/eje.12629>.

[6] K. Asimakopoulou, A. Gupta, S. Scambler, Patient‐centred care: barriers and opportunities in the dental surgery, Community Dent Oral Epidemiol. 42 (2014) 603–610. <https://doi.org/10.1111/cdoe.12120>.

[7] M.J. Nowak, H. Buchanan, K. Asimakopoulou, ‘You have to treat the person, not the mouth only’: UK dentists’ perceptions of communication in patient consultations, Psychol Health Med. 23 (2018) 752–761. <https://doi.org/10.1080/13548506.2018.1457167>.

[8] J. Cane, D. O’Connor, S. Michie, Validation of the theoretical domains framework for use in behaviour change and implementation research, Implementation Science. 7 (2012) 37. <https://doi.org/10.1186/1748-5908-7-37>.
